# Modular GAN: positron emission tomography image reconstruction using two generative adversarial networks

**DOI:** 10.3389/fradi.2024.1466498

**Published:** 2024-08-29

**Authors:** Rajat Vashistha, Viktor Vegh, Hamed Moradi, Amanda Hammond, Kieran O’Brien, David Reutens

**Affiliations:** ^1^ Centre for Advanced Imaging, University of Queensland, Brisbane, QLD, Australia; ^2^ ARC Training Centre for Innovation in Biomedical Imaging Technology, University of Queensland, Brisbane, QLD, Australia; ^3^ Diagnostic Imaging, Siemens Healthcare Pty Ltd., Melbourne, QLD, Australia

**Keywords:** PET image reconstruction, deep learning, generative adversarial network, noise and motion correction, non-clinical training data

## Abstract

**Introduction:**

The reconstruction of PET images involves converting sinograms, which represent the measured counts of radioactive emissions using detector rings encircling the patient, into meaningful images. However, the quality of PET data acquisition is impacted by physical factors, photon count statistics and detector characteristics, which affect the signal-to-noise ratio, resolution and quantitative accuracy of the resulting images. To address these influences, correction methods have been developed to mitigate each of these issues separately. Recently, generative adversarial networks (GANs) based on machine learning have shown promise in learning the complex mapping between acquired PET data and reconstructed tomographic images. This study aims to investigate the properties of training images that contribute to GAN performance when non-clinical images are used for training. Additionally, we describe a method to correct common PET imaging artefacts without relying on patient-specific anatomical images.

**Methods:**

The modular GAN framework includes two GANs. Module 1, resembling Pix2pix architecture, is trained on non-clinical sinogram-image pairs. Training data are optimised by considering image properties defined by metrics. The second module utilises adaptive instance normalisation and style embedding to enhance the quality of images from Module 1. Additional perceptual and patch-based loss functions are employed in training both modules. The performance of the new framework was compared with that of existing methods, (filtered backprojection (FBP) and ordered subset expectation maximisation (OSEM) without and with point spread function (OSEM-PSF)) with respect to correction for attenuation, patient motion and noise in simulated, NEMA phantom and human imaging data. Evaluation metrics included structural similarity (SSIM), peak-signal-to-noise ratio (PSNR), relative root mean squared error (rRMSE) for simulated data, and contrast-to-noise ratio (CNR) for NEMA phantom and human data.

**Results:**

For simulated test data, the performance of the proposed framework was both qualitatively and quantitatively superior to that of FBP and OSEM. In the presence of noise, Module 1 generated images with a SSIM of 0.48 and higher. These images exhibited coarse structures that were subsequently refined by Module 2, yielding images with an SSIM higher than 0.71 (at least 22% higher than OSEM). The proposed method was robust against noise and motion. For NEMA phantoms, it achieved higher CNR values than OSEM. For human images, the CNR in brain regions was significantly higher than that of FBP and OSEM (*p* < 0.05, paired *t*-test). The CNR of images reconstructed with OSEM-PSF was similar to those reconstructed using the proposed method.

**Conclusion:**

The proposed image reconstruction method can produce PET images with artefact correction.

## 1 Introduction

The reconstruction of PET images transforms a sinogram, representing counts of radioactive emissions measured in rings of detectors placed around the patient, into images. Analytical and iterative reconstruction methods based on mathematical models that represent imaging systems have been widely used in commercial scanners. For example, early reconstruction algorithms such as filtered backprojection (FBP) utilised the Radon transform, and incorporated corrections to account for random noise, attenuation and scatter (1). The transition from analytical to iterative methods improved image quality by explicitly incorporating domain knowledge of imaging physics in more sophisticated mathematical models. With these methods, overfitting may cause artefacts and noise in the reconstructed images (2), necessitating regularisation strategies which increase the complexity and computational load of reconstruction and sometimes require additional information from other imaging modalities (3).

Recently, Deep Learning (DL) neural networks have been applied to image reconstruction, with the goals of improving spatial resolution and signal-to-noise ratio with lower scan time and injected dose. The state-of-the-art DL methods for PET image reconstruction have recently been reviewed (4, 5). One application of DL has been to correct errors after iterative or analytical image reconstruction (3, 6, 7). Recent studies have adopted the strategy of incorporating neural networks within an iterative image reconstruction framework (8). Incorporating neural networks into iterative processes enhances reconstruction quality but requires experimental fine-tuning of the hyperparameters (9). Mehranian and Reader introduce a model-based deep reconstruction network for data-driven hyperparameter adaptation (10) to enable parameter sharing, thus reducing the number of trainable parameters. In a different method deep image prior was used in an unsupervised manner for sinogram to PET image reconstruction using a forward projection model (11). The results of these studies are promising, however the strategy inherits the complexity of iterative methods in which multiple forward and backward projections and modelling of imaging systems are required (10). In addition, iterative reconstruction with deep learning falls short with respect to total reconstruction time, whereas direct data-driven models once trained adequately, have the potential for rapid, high quality image reconstructions (with remarkable, up to 36-fold, reductions in reconstruction time) compared to traditional iterative algorithms (12).

Direct data-driven reconstruction methods do not rely on iterative models and ignore the physics underpinning the imaging modality. They do not require explicit modeling of imaging systems. However, their performance does depend on a comprehensive and diverse training dataset. Automated Transform by Manifold Approximation (AUTOMAP) established the feasibility of purely data-driven approaches with convincing results demonstrated for MRI reconstruction (13). Reconstructed PET images were unable to resolve smaller anatomical structures well, perhaps because the network used for PET reconstruction was trained using the 2D Radon transform of MRI data, resulting in a mismatch between the resolution of the training and test data set (14). The structure of the neural network structure required large computational memory, particularly for large images, because raw data were fully connected to the dense layer.

DeepPET utilised high resolution PET training data, thereby obviating resolution mismatch (15). An encoder-decoder network was used, with the convolution layer connected to the raw input data, reducing memory requirements compared to AUTOMAP. The encoder network transformed input sinograms to a learned latent space, a representation of limited spatial sampling of sinogram features. The PET image was then reconstructed by progressive deconvolution of the latent space information by a decoder network. PET images reconstructed using DeepPET were blurred and lacked fine detail (14), motivating the development of methods such as LAFOV-PET utilising perceptual loss functions. Perceptual losses reflect the difference between generated and target images in features such as texture (spatial intensity variations) or edges (contours with abrupt changes in intensity), object shape or patterns in the image (16). Despite this, LAFOV-PET did not produce high-quality images when large anatomical distortions were present in patient images or when non-anatomical objects such as the NEMA phantom were imaged, highlighting the dependence of the method's performance on information in the training data (12).

Generative adversarial networks (GAN) require substantially smaller training datasets than previously used DL methods. During training, generated images are quantitatively evaluated by an adversary network (discriminator) to minimise the difference between the real and the generated image (17). GANs allow implicit learning of the underlying feature distribution in the training data and, once trained, generate images using the learned distribution (18). In comparison, encoder-decoder based methods explicitly learn the internal representation of training data by minimising pixel-wise error. A GAN based method for direct sinogram to image reconstruction, DPIR-NET, required fewer training images than LAFOV-PET and produced high quality images. Validation studies only addressed small-scale variability in the test set, raising questions about overfitting to application specific training data (12). A consequence of overfitting is that if training using images from normal subjects may not enable accurate reconstruction of images from patients with structural abnormalities due to pathology.

In this paper, we propose a GAN that learns the sinogram to image translation from a limited number of synthetic training images. We explore the properties of training images that prevent overfitting by the GAN to a specific image type when non-field specific training images are used. The proposed methodology comprises two distinct modules. Module 1 is designed following the Pix2pix-HD architecture and is trained using pairs of non-clinical sinograms and images (19). Module 2 aims to perform image to image translation with the aim to enhance image quality without the need for additional clinical images. Here, we utilise a Style GAN framework by using an additional mapping network to generate style-regulating parameters (20). This additional mapping network transforms the encoded input representations into an intermittent latent code. This intermittent latent code, when subjected to an affine transformation, governs styles through adaptive instance normalisation (AdaIN). For localised style transfer, AdaIN first normalizes and then scales and shifts the feature maps based on the style-regulating parameters. As a last step, the synthesis network generates an image using the transformed latent code. We compared the performance of the proposed direct reconstruction method against existing algorithms (FBP, OSEM and OSEM-PSF) and assessed its ability to correct errors due to signal attenuation, patient motion and low count/noise in the acquired data.

## 2 Method

### 2.1 PET image reconstruction

**Figure 1:**
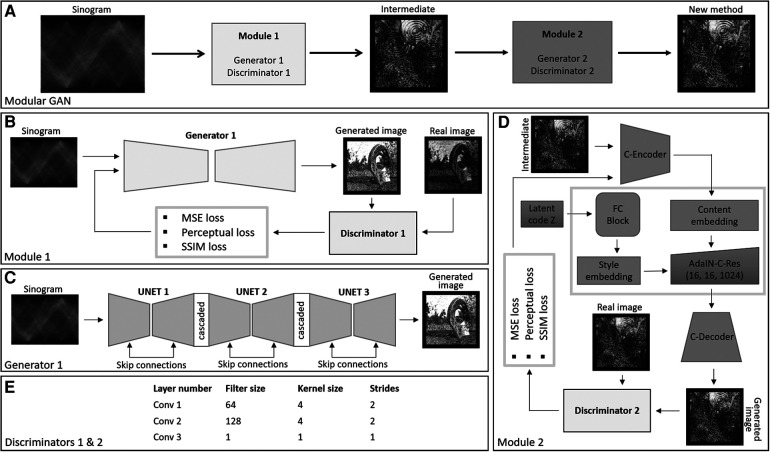
**(A)** The figure illustrates the framework of the modular GAN. It comprises two modules: Module 1 transforms the sinogram into a coarse-grained image. The output of Module 1 serves as input for Module 2, another GAN that performs fine-grain image enhancement. **(B)** Training process of the GAN. The generator and discriminator engage in an adversarial process, competing with each other using three different types of losses. **(C)** Represents the architecture of the generator for Module 1 and **(D)** represents the architecture of the generator for Module 2. **(E)** Shows the receptive field of 10 for discriminators used to train the generators of two modules.

We propose the use of a modular deep learning method consisting of two GANs. The first GAN transforms the sinogram to a coarse-grained image, and the second GAN takes the output of the first GAN and performs fine-grain image enhancement. The GANs are based on the Pix2Pix and style GAN frameworks, respectively (refer to Figure 1).

#### 2.1.1 Module 1—Pix2Pix GAN

We used the conditional GAN proposed in (19) as the base model for this part of the deep learning framework. The generator here is a cascaded U-Net (21), and the discriminator is based on patchgan (18). The input to the U-Net is the PET sinogram, here considered to be of dimension 256 × 256 × 3, where the last dimension is produced by replicating the sinogram three times to satisfy the VGG-19 input condition. The encoder part of the U-Net involved eight convolutional layers followed by instance normalisation and leaky-ReLU activation. The first layer had 64 filters of kernel size 4 × 4 and stride 2. Subsequent layers had the same kernel size, and filter sizes were set to 128, 256, 512, 512, 512, 512 and 512. The bottleneck layer had ReLU activation without normalisation. Similarly, the decoder consisted of eight layers of transpose convolution with filters in reverse order and the same kernel size and stride as the encoder. The decoder output layer incorporated the Tanh activation function. The discriminator network was based on patchgan, consisting of convolution layers having 64, 128, filters followed by instance normalisation and leaky-ReLU activation. The kernel size was set to 4 × 4 with a stride of 2. The last layer involved a filter size, kernel size and stride of 1.

#### 2.1.2 Module 2—style GAN

Style GAN involves an encoder, a mapping network and a synthesising network (C-Decoder). Here, we have added an encoder, called the C-Encoder, as the first step. The C-Encoder consisted of convolutional layers of filters of size 64, 128, 256, 512 and 512, and kernel size of 4 × 4 and stride of 2, except the 64-filter layer which had a stride of 1. The last layer was followed by three residual blocks, consisting of two 512 filer size layers involving instance normalisation and ReLU activation with kernel size of 4 × 4 and stride of 1. To form the residual connection, the output prior to the residual blocks was concatenated with the output of the second residual block convolutional layer. The C-Encoder is used to extract the hierarchical features from the images generated using Module 1. The use of two-stride convolution layers enables neural style transfer and super resolution (22). The mapping network, FC-Block consisted of 3 fully connected layers followed by instance normalisation and ReLU activation. The dimension of each fully connected layer was kept the same, corresponding to the filter size of the style embedding layer. Latent noise of dimension 1,024 was passed to the FC-Block.

The output of the C-Encoder was embedded with the FC-Block output using AdaIN blocks (20). Then AdaIN block consisted of the two convolution layers followed by adaptive instance normalisation and ReLU activation, added using skip connections. The C-Decoder consisted of transpose convolutional layers having 512, 256, 128, 64 filters and kernel size of 4 × 4 and stride of 2. All transpose convolutional layers were followed by an instance normalisation and Relu activation, while 512, 256 and 128 layer constitutes dropouts between normalisation and activation. The last layer of the decoder was a convolutional layer with kernel size 4 × 4, filter size 3 and stride of 1. Module 2 discriminator was patchgan based with 64 and 128 filters for the convolutional layers, followed by instance normalisation and leaky-ReLU. The kernel size was set to 4 × 4 with a stride of 2. The last layer involved a filter size, kernel size and stride of 1.

### 2.2 Adversarial and non-adversarial loss functions

Adversarial loss and non-adversarial losses were implemented in both Module 1 (M1) and Module 2 (M2) (19). The M1 adversarial loss function for the discriminator output was defined as:

(1) $$L_{M1,adv}=E_{s,t}\{\log D(s,t)\}+E_{s}\{\log(1-D(s,\;G(s)))\},$$

where, *D(s, t)* and *D*[*s, G(s)*] denote the outputs of the discriminator for real and fake outputs respectively, t' [*t*' = *G*(*s*)] denotes the image generated from *s* and *E* denotes mathematical expectation. $L_{M2,adv}$ is of the same form as (1), but *s* is replaced by *t'*, the output of M1 and *t"* [*t"= G(t', z)*] is the output of M2, where *z* is the latent noise. Notably, M1 involves domain transformation and we did not explicitly add *z* in M1 as the dataset itself contains enough variance, whilst M2 performed the task of image-to-image enhancement and z is added in the generator to involve stochastic transformation for corrections. The M1 pixel reconstruction loss is defined as:

(2) $$L_{M1,pix}=E_{s,t}\{t^{\prime }-t\},$$

where for $L_{M2,pix}$, *s* is replaced by *t*'. Wang et al. concluded that the need for an additional network for adversarial perceptual loss can be eliminated by using a discriminator as a trainable feature extractor (19):

(3) $$L_{M1,perc}=\;\overset{L}{\underset{n=1}{\sum }}w_{p}\frac{1}{N_{n}}\{E_{s}[D_{n}(s,\;G(s))]-E_{s,t}[D_{n}(s,t)]\},$$

where, L is the number of hidden layers in the discriminator, *D_n_* defines the feature representations extracted from the n^th^ hidden layer of the discriminator, $w_{c\;}=1/2,$ is the weighting of the contribution of each convolutional layer of the discriminator and *N_n_* is the number of elements in each layer. $L_{M2,perc}$ has the same form as (3), but *s* is replaced by *t*'. Style and content transfer non-adversarial loss were adopted from a neural style transfer framework using VGG-19 as the pretrained network. Style loss was incorporated by calculating the Frobenius squared norm:

(4) $$L_{M1,style}=\;\overset{B}{\underset{i=1}{\sum }}w_{s\;}\frac{1}{4d_{i}^{2}}Gram_{i}(t^{\prime })-Gram_{i}(t{)}_{F}^{2},$$

where, B is the number of convolutional blocks used from the pretrained VGG-19, $w_{s\;}=1/5,$ is the weighting of the contribution of each convolutional block, d is the spatial depth, $Gram_{n}(t)\;$ and $Gram_{n}(t^{\prime })$ are the Gram matrices that represent the feature correlations of each convolutional block (*V_i_*) for the target image and generated image, respectively. $L_{M2,style}$ has the same form as (4), but *t'* is replaced by *t'’*. The content loss was calculated using:

(5) $$L_{M1,content}=\;\overset{B}{\underset{i=1}{\sum }}w_{c\;}\frac{1}{N_{i}}V_{i}(t^{\prime })-V_{i}(t{)}_{F}^{2},$$

where, B is the number of convolutional blocks, $w_{c\;}=1$, is the weighting of the contribution of each convolutional block, *N_i_* is the number of elements in each layer, $V_{i}(t)\;$ and $V_{i}(t^{\prime })$ are the feature maps extracted for each convolutional block for the target image and generated image, respectively. $L_{M2,content}$ is of the same form as (5), but *t'* was replaced by *t"* (23).

To measure the global similarity between the target image and reconstructed image, we used a previously proposed structural similarity loss function:

(6) $$L_{M1,ssim}=1-SSIM(t,t^{\prime }),$$

and for $L_{M2,ssim}$ *t'* is replaced by *t"*. The holistic loss function is the weighted sum of all the loss functions as depicted in Equations 1–6, including adversarial and non-adversarial losses:

$$L_{M1}=L_{M1,adv}+w_{1}L_{M1,pix}+w_{2}L_{M1,perc}+w_{3}L_{M1,style}+w_{4}L_{M1,cont}+w_{5}L_{M1,ssim},$$

where weights *w*_1_ to *w*_5_ scale the contribution of non-adversarial losses to the overall M1 loss function. Here, we set weights to 1, 10, 0.0001, 0.0001 and 5, respectively. $L_{M2}$ is formed similarly to $L_{M1}$.

### 2.3 Training dataset

Most GAN studies derive training and testing data from a single type of data source, whereas we chose to create a training dataset independent of the testing data. The training data were derived from synthetic images (24–27) and testing was performed on acquired real human brain images. A detailed description of training dataset features is provided in Supplementary Material-1, including measures of entropy, symmetry, contrast and fractal dimension (28–31). These metrics were used to characterise the optimal training dataset. The training dataset providing the best performance was used to train the intermediate module. The synthetic training images can be accessed via a supplied link. To train Module 2 only, synthetic PET brain images, generated using an atlas MRI, were used in addition to synthetic non-PET images with fractal dimensions and entropy resembling that of brain images. In addition, data augmentation (including flipping, rotation, wrapping, intensity adjustments, blurring, and cropping) were applied to the synthetic PET brain images, before training Module 2 (32, 33).

### 2.4 Testing dataset—simulated brain images

For test data, anatomical MR images of the brain from various participants were utilised. These images were obtained from an open-source medical imaging repository known as Brainweb (34). These were used to generate synthetic FDG-PET images by simulating tissue tracer uptake with the two-compartment irreversible uptake model of FDG metabolism. To generate the PET image, tracer uptake was modelled using kinetic parameters for voxels in four tissue classes were segmented from the MRI into gray and white matter, vessels, and tissue around the brain fat (35). Uptake values for each of the tissue types were set to match the contrast of a typical 18F-FDG PET scan, with uptake in the gray matter being four times greater than the uptake in the white matter. The voxel size of the resulting simulated brain test data was 1 × 1 × 1 mm^3^ with a matrix size of 256 × 256. A smoothing kernel based on the effective resolution of a clinical scanner was not applied.

In the testing data set, we simulated lesions with high FDG uptake by randomly placing circular lesions with higher tracer uptake in different regions of the brain. Axial, sagittal and coronal PET image slices were converted to noise free sinograms (representing high true counts) using the Radon transform in a PET simulator (36). These simulated synthetic FDG-PET images were used as ground truth images to test the performance of the proposed proposed method against FBP and OSEM. dPETSTEP was used for FBP and OSEM reconstructions (36). In addition, structural similarity (SSIM), peak signal to noise ratio (PSNR) and relative root mean squared error (rRMSE) were used to quantitatively compare the image reconstruction methods (16).

The image matrix size of training and testing images was 256 × 256. Before being used as inputs to the network, source and target images were normalised to −1–1 according to the nomenclature used in Pix2Pix GAN (18). Sinograms of training and test images were generated to match the input of the network. Sinograms and source images were converted to 3 channels, replicating the last dimension by multiplying it, using the tile function to enable use with the pre-trained VGG-19 network to evaluate style transfer losses.

#### 2.4.1 Artefact simulation

The proposed method was assessed against noise, motion and attenuation-based artefacts. To simulate motion, synthetic PET brain images (ground) were subjected to rotation with three different angles: 2°, 5°, and 10°. The radon transform of the original ground image and the subsequently rotated images were weighted and added together, as depicted in Supplementary Figure 3s-A. To simulate low true counts and assess the impact of noise, Poisson noise of varying magnitudes was applied to the sinogram of the ground truth image. The resulting noisy sinograms were utilised as inputs and compared with the known ground truth. To assess the impact of attenuation, smaller circular cavities and a wedge section from the white and grey matter of the ground PET brain images were removed. Radon transformations of the resulting images were then used as input. The removal of parts of the image enabled us to evaluate the ability of the proposed method to accurately reconstruct the image taking into account spatial attenuation effects.

### 2.5 Validation dataset

#### 2.5.1 NEMA phantom

A whole body NEMA phantom was scanned on a Biograph Horizon (Siemens Healthineers) scanner. The phantom consists of four parts in a solid polyethylene cylinder—the background body, six fillable spheres with internal diameters of 10, 13, 17, 22, 28 and 37 mm, a non-radioactive cylindrical insert in the centre of the phantom and a line source (37). F-18 was used to fill the phantom background and the four small spheres with an activity concentration ratio of 1:8 (background: spheres). The two largest spheres were filled with water only. The non-radioactive cylindrical insert was placed in the centre of the phantom. The line source used to simulate scatter fraction, count losses and random measurement was injected with 110 MBq of activity to yield an effective activity concentration equal to the background.

The whole phantom was scanned for 20 min. The percentage contrast recovery was calculated by comparing the measured activity concentration in each sphere to the known true activity concentration as defined by the NEMA standards (37). In addition, the contrast-to-noise ratio (CNR) was calculated specifically for the spheres filled with activity (38):

(7) $$\;\;\;CNR=\frac{\;ROI\;i-\;background\;i}{\;\;\;\;\mathrm{standard}\;\mathrm{deviation}\;\mathrm{of}\;\mathrm{the}\;\mathrm{background}\;\mathrm{ROI}\;\mathrm{counts}},$$

where *ROI i* denotes average counts in the region of interest (ROI) for sphere and *background i* denotes the average counts in a ROI placed in a uniform area outside the spheres.

#### 2.5.2 Human dataset

Approval for this project was granted by the Human Research Ethics Committee of the University of Queensland (2021/HE001605). Written consent was obtained from the five healthy male participants. List mode acquisition using the Biograph Horizon PET scanner (Biograph Horizon 3R-VJ21C) at the Centre for Advanced Imaging, University of Queensland, was started 15 s prior to the intravenous bolus injection of −200 MBq of ^18^F-FDG followed by a 50 ml saline flush. Total acquisition time was 60 min.

##### 2.5.2.1 Qualitative and quantitative comparison

47–59 min list mode data were used to compare FBP, OSEM, OSEM-PSF and the proposed method. Standard scanner-based corrections were applied for time-of-flight, normalisation, gap filling, attenuation, scatter and random. Two-dimensional Fourier rebinned sinograms, obtained using the investigational software prototype e7 tools (Siemens Healthineers), were of dimension 140 × 360 (angular and radial bins) (23). Supplementary Figure 7s depicts the steps followed to ensure compatibility of the acquired scanner data with the input training data having dimension 256 × 256. The sinograms were flipped vertically and then horizontally concatenated with the original sinograms to increase the sampling of projection angles from 180° to 360°. The resultant sinograms were centrally cropped to eliminate void spaces. The cropped sinograms were normalized to make them compatible with the training data. The images reconstructed by the proposed method were qualitatively compared with FBP, OSEM and OSEM-PSF reconstructions implemented on the scanner. The axial image matrix size was set to 256 × 256, resulting in an image resolution of 2.89 × 2.89 × 2.02 mm^3^.

To appreciate the differences between FBP, OSEM, OSEM-PSF and the proposed method, we performed a second level quantitative analysis on all participants. Six brain regions (caudate, lentiform nucleus, cerebellum, parietal lobe, frontal gray matter and temporal lobe) were considered for contrast-to-noise ratio analysis, with respect to the background taken from the genu and splenium regions of white matter (refer Equation 7).

##### 2.5.2.2 Validation against artefacts

**Figure 2:**
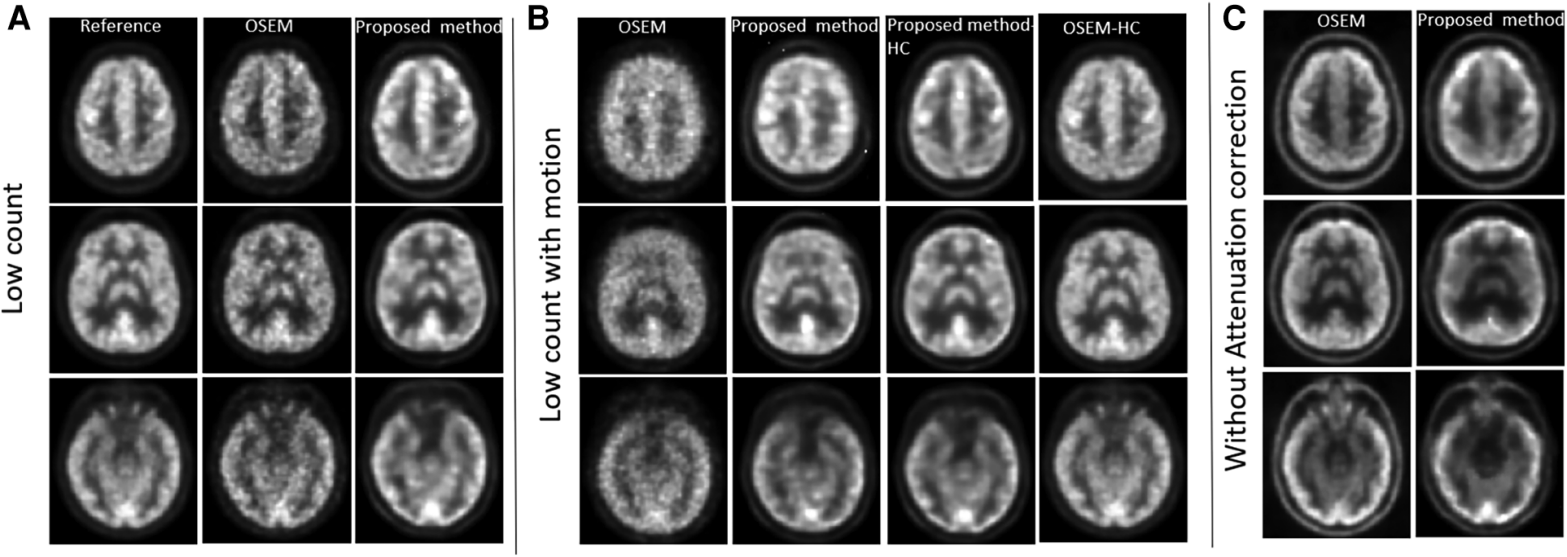
PET reconstruction qualitative comparisons between OSEM and proposed method against corrections. **(A)** Influence of using low count sinogram data as input in Modular GAN is assessed. **(B)** Low count data acquired in the presence of extensive participant motion is analysed and compared with high count motion images. **(C)** Impact of not using attenuation correction for the input sinogram data.

To validate the proposed method against motion, noise and attenuation, data from different time frames were used. To assess motion, participants were asked to move their head slightly during the last minute of the 60 min list mode acquisition. To assess the impact of noise, we generated sinograms and images using data corresponding to low count (53–54 min). To test against noise with motion, we used low count data with motion (59–60 min) and high count data with motion (53–60 min). In contrast, to compare reconstructed images, we utilised data from high count and without motion as reference (53–59 min, Figure 2A). The same high count time frame was also used to test the impact of not using attenuation correction in the input sinogram.

### 2.6 Statistical analysis

The differences in SSIM, PSNR, rRMSE (for simulated data) and CNR (for NEMA phantom and human data) between the proposed method, FBP and OSEM, were evaluated for statistical significance using the paired *t*-test. The threshold for statistical significance was set at *p* < 0.05 (one-tailed test). To confirm the normality assumptions, the Shapiro-Wilks test was used at a significance level of *p* < 0.05 (39).

### 2.7 Comparative analysis

The Module 1 of proposed method has been compared with the DeepPET and image conditional GAN trained using the non-clinical images. We have used the original architecture as proposed in DeepPET (15) and conditional GAN, lacking the use of perceptual or style transfer-based loss functions (18).

## 3 Results

Results are provided for synthetically created test images, followed by *in vivo* human PET experiments. The results have been arranged in a way which allows verification of the Modular GAN components in Figure 1.

### 3.1 Module 1—sinogram to intermediate image mapping using simulated data

**Figure 3:**
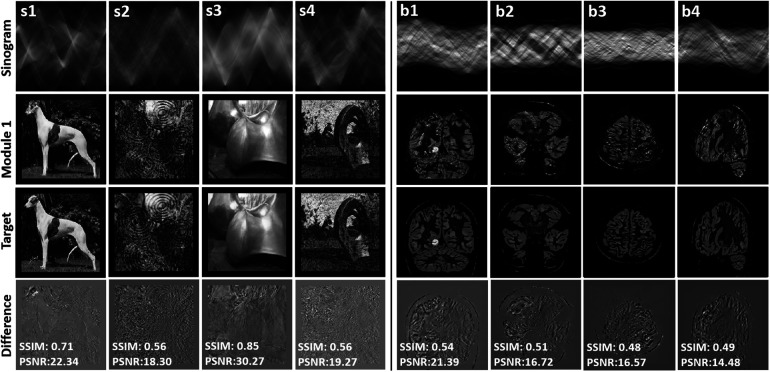
The figure presents the results of the reconstructed synthetic training (s1–s4) and testing images (b1–b4) using only module 1 GAN. The top row displays the input sinogram provided as input to the GAN. The third row showcases the ground truth images, representing the ideal reference images for comparison. In the second row, the reconstructed images obtained from Module 1 GAN are displayed. The fourth row exhibits the difference images, highlighting the variations and discrepancies between the ground truth and reconstructed images. SSIM and PSNR metrics quantifying the similarity and quality of the reconstructed images in comparison to the ground truth. This evaluation allows for a comparison against ground truth images.

Figure 3 presents the reconstructed synthetic output images of Module 1 (refer to Figure 1B). Examples denoted by s1–s4 correspond with the training of Module 1, and b1–b4 are unseen synthetic brain images used for testing. Module 1 maps a sinogram to the intermediate image. The intensity difference between the intermediate image and the target image is provided to appreciate the quality of the mapping achieved by Module 1. SSIM and PSNR are provided as a measure of how well the sinogram has been mapped to the target image. The achieved SSIM between intermediate and target images was between 0.56 and 0.85, providing an output with reasonable structural similarity and intermediate image quality sufficient to discern object detail.

In the Supplementary Figure 5-s, Module 1 of the proposed method has been compared with two direct PET image reconstruction methods when non-clinical images were used for training. A qualitative examination of the results reveals that deepPET produces images with increased blurriness and fewer details. The conditional GAN, which does not use perceptual or style transfer-based loss functions, generates images of better quality than deepPET. Both deepPET and the conditional GAN yield inferior images to those generated by Module 1.

### 3.2 Module 2—intermediate image to final image mapping using simulated data

#### 3.2.1 Qualitative and quantitative comparison

**Figure 4:**
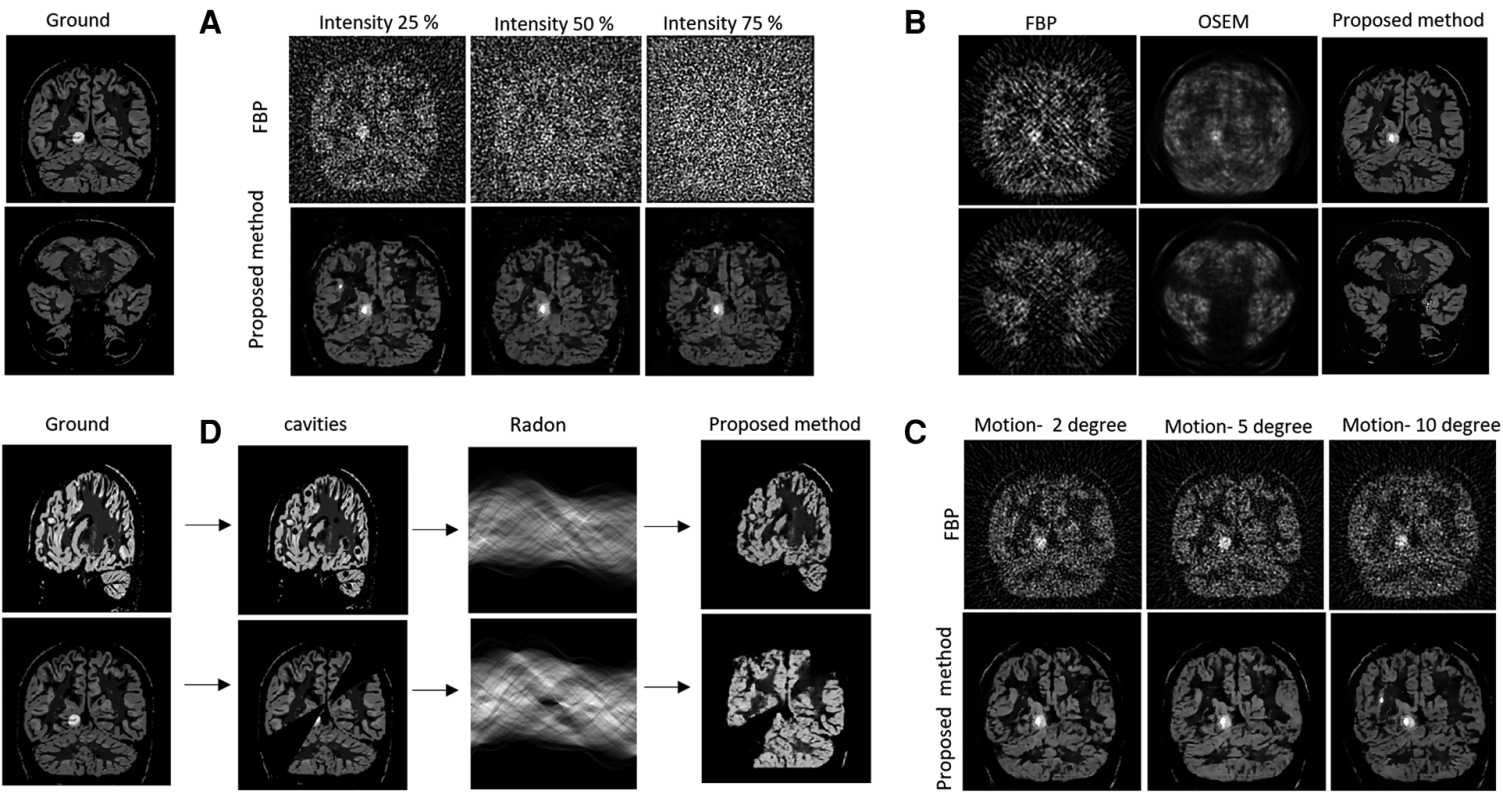
Ground truth images are displayed at left. (**A**) Validation against noise. Top row represents the filter back projected images of the noisy sinograms (with varied noise intensities) and bottom row represents the image reconstructed using proposed method. **(B)** Comparison of image quality for noisy sinogram when reconstructed with FBP, OSEM and proposed method. **(C)** Noise and motion with simulated head degree reconstructed using the FBP and proposed method. **(D)** Cavities forward projected using Radon transform and reconstructed using the proposed method.

Figure 4 provides the test results for the simulated data. The output of Module 2 is labelled as “Proposed method”. The ground is the simulated subject image from Brainweb, without the use of standard smoothing filters applied in PET reconstruction. It can be seen in Figure 4B that the proposed method produced higher quality images than FBP and OSEM. This is quantitatively confirmed by the SSIM, PSNR and rRMSE values provided in Table 1 for four example brain images (b1–b4). The differences in SSIM, PSNR and rRMSE between the proposed method and FBP were found to be statistically significant (*p* = 0.0001, 0.0022 and 0.0004, paired *t*-test). Similarly, the differences in PSNR and rRMSE between the proposed method and OSEM were also significant (*p* = 0.02 and 0.002). However, the difference in SSIM between OSEM and the proposed method (*p* = 0.06) was not statistically significant.

**Table 1:** Table analyse the SSIM, PSNR and rRMSE between the test ground image and images (Figure 3, b1–b4) reconstructed using FBP, OSEM and proposed method.

Method	SSIM-b1	SSIM-b2	SSIM-b3	SSIM-b4	PSNR-b1	PSNR-b2	PSNR-b3	PSNR-b4	rRMSE-b1	rRMSE-b2	rRMSE-b3	rRMSE-b4
FBP	0.30	0.31	0.29	0.30	16.47	13.16	13.89	13.39	1.15	1.42	1.76	1.44
OSEM	0.58	0.62	0.75	0.69	20.70	17.17	17.64	17.31	0.70	0.89	1.14	0.91
Module 1	0.54	0.51	0.48	0.49	21.39	16.72	16.57	14.48	0.77	1.01	1.38	1.13
Proposed method	**0** **.** **78**	**0**.**77**	**0**.**78**	**0**.**71**	**22**.**97**	**19**.**65**	**19**.**83**	**17**.**62**	**0**.**48**	**0**.**72**	**0**.**85**	**0**.**74**

FBP, OSEM and Module 1.

Bold values indicates that the proposed method outperformed.

#### 3.2.2 Validation against noise, motion, and cavities

Figure 4A shows the effects of injecting different levels of Poisson noise into the sinogram, Figure 4B provides results for low count sinograms, Figure 4C provides the simulated motion result and Figure 4D depicts the effects of cavities (i.e., spatial inconsistencies in images). Qualitatively, the figures provide strong evidence for the robustness of the proposed method to both noise and motion. They show improved contrast and a reduction in motion and noise-based artefacts when compared to FBP. In Figure 4D, the observed partial recovery of missing data in the cavities, but not in the wedges, illustrates the spatial consistency of the intensity projection from sinogram to the image.

**Figure 5:**
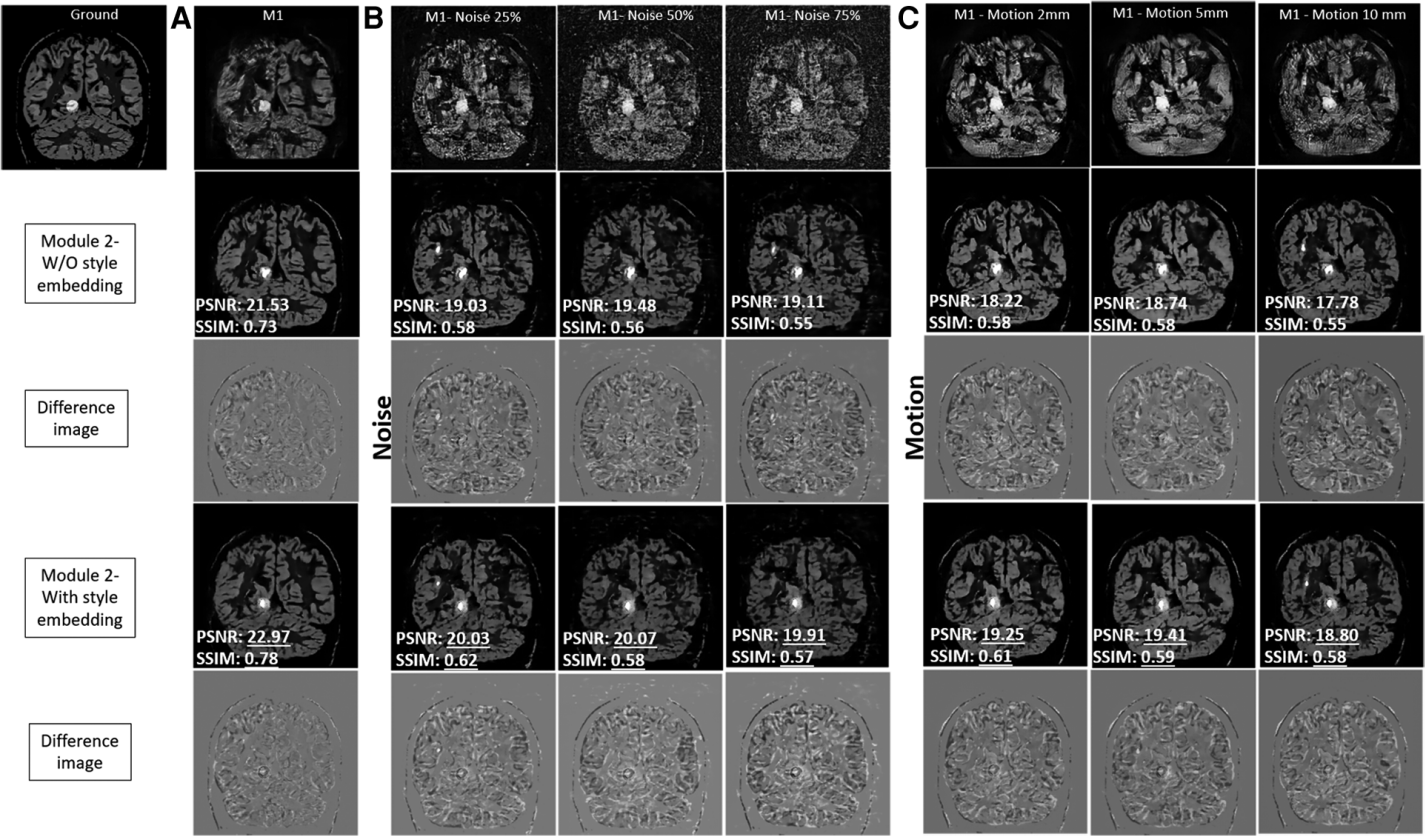
Comprehensive analysis of the output produced by module 1 and module 2 for the test brain images. The results produced using style embedding within the generator of Module 2 are also provided. The two modules have been compared in presence of no noise **(A)**, against simulated varying noise intensities **(B)** and different head motion **(C)** to validate performance without and with style embedding.

Figure 5 provides an evaluation of the output produced by Module 1 and Module 2 for the test brain images. The results produced using style embedding within the generator are also provided. In Figure 5A, in the absence of noise, Module 2 with and without the use of style embedding produces images with high contrast, effectively enhancing images produced by Module 1. To appreciate the quality of the mapping achieved by Module 2 with style embedding, the intensity difference between Module 2 variants and the ground truth image is provided, reflecting around 5% improvement in SSIM and PSNR values. Figures 5B,C explore the case of varying noise intensities and different head motions. The quantitative evaluation suggests improvements in PSNR and SSIM with style embedding in Module 2, compared to when style embedding is not used. However, the amount of improvement was found not to be statistically significant.

### 3.3 Trained modular GAN tested using NEMA phantom

**Figure 6:**
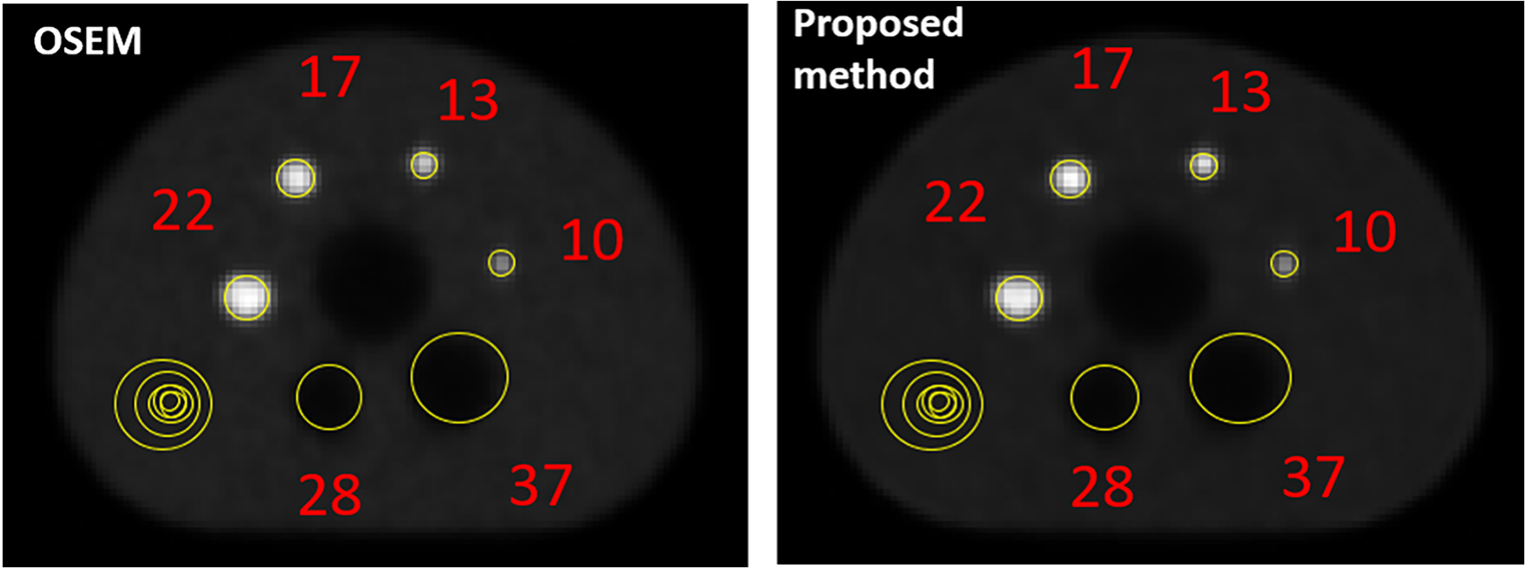
NEMA image quality analysis for the image reconstructed using OSEM and developed proposed method.

Figure 6 provides images of the phantom reconstructed using OSEM and the proposed method. Table 2 quantifies the image quality for each case measured by the contrast recovery (%) and contrast-to-noise ratio. The image reconstructed using the proposed method appears to be less noisy than that obtained using OSEM. On average, the proposed method achieved around 10% larger contrast recovery and 21% larger contrast-to-noise ratio for hot spheres than OSEM. For all hot and cold spheres, the proposed method achieved higher contrast recovery (*p* = 0.01) and contrast-to-noise ratios (*p* = 0.003) than OSEM.

**Table 2:** Comparison between OSEM and proposed method using NEMA standards. Table presents contrast recovery percentage and contrast-to-noise ratio for hot spheres with diameter of size 10, 13, 17 and 22 and cold spheres with diameter of size 28 and 37.

Sphere diameter (mm)	Contrast recovery % OSEM	CNR OSEM	Contrast recovery % Proposed method	CNR Proposed method
Hot spheres				
10	31	43	39	54
13	47	71	48	92
17	70	74	73	87
22	74	104	79	118
Cold spheres				
28	62	–	63	–
37	67	–	71	–

### 3.4 Trained modular GAN tested using human PET data

#### 3.4.1 Qualitative and quantitative comparison

**Figure 7:**
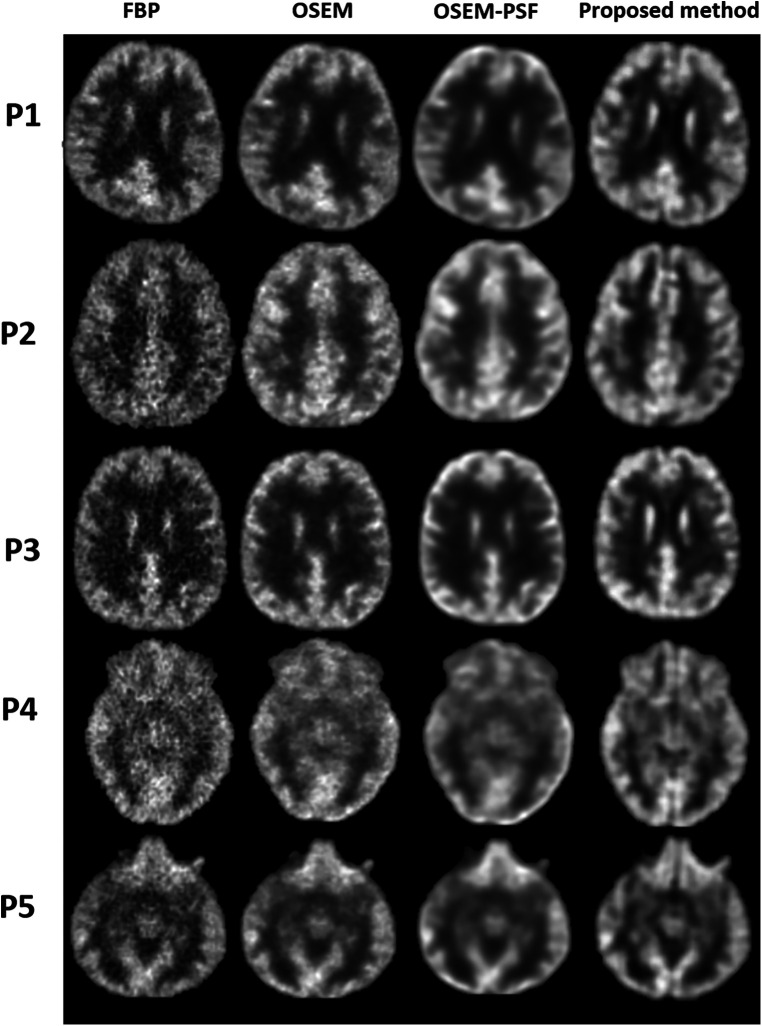
Qualitative comparison between the images reconstructed using FBP, OSEM, OSEM-PSF and proposed method has been shown for five human participants (P1–P5).

Figure 7 compares the images reconstructed using FBP, OSEM, OSEM-PSF and the proposed method for acquired human data. Qualitative inspection reveals that the proposed method surpasses FBP and OSEM in performance. Specifically, the proposed method reduced noise, enhanced contrast and improved depiction of structures compared to FBP and OSEM. Additionally, OSEM-PSF tended to blur smaller anatomical structures, while the proposed method provided a clear depiction of these structures. Notably all images for P1, and others, have differences amongst them. The proposed method produces images with the clearest level of detail, particularly evident for cortical regions. This may be due to the fact that the point-spread-function defines the apparent resolution limit of the OSEM and OSEM-PSF reconstruction. However, the proposed method relies on the MRI atlas, which according to our results, is beneficial for improving the level of detail in reconstructed images.

**Figure 8:**
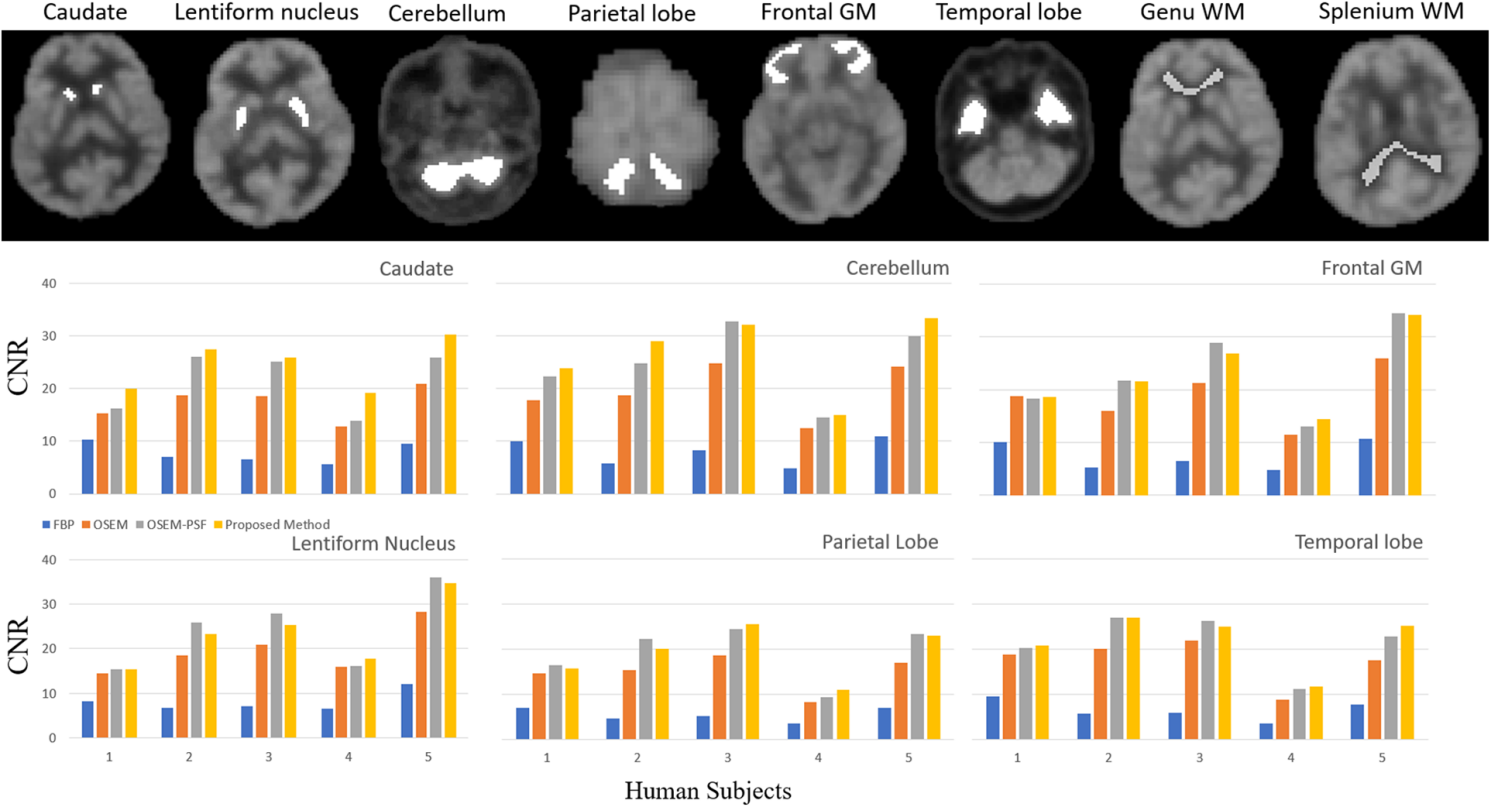
Quantitative comparison of the contrast-to-noise ratio of brain cortical regions represented at the top of the figure (caudate, lentiform nucleus, cerebellum, parietal lobe, frontal gray matter and temporal lobe, background from genu and splenium of white matter). The bar graphs show that for the five human subjects in each region the OSEM (with and with-out PSF) method outperforms FBP, and the proposed method also outperforms FBP and OSEM. Insignificant difference was observed between proposed method and OSEM-PSF.

In Figure 8, the bar graphs show that for each region, OSEM (with and without PSF) outperformed FBP, and the proposed method outperformed both FBP and OSEM. However, the contrast-to-noise ratio achieved by the proposed method and OSEM-PSF were similar. Table 3 summarises the results of statistical analysis of these findings. The proposed method provided significantly larger contrast-to-noise ratios compared to FBP and OSEM (*p* < 0.05) for all regions defined in Figure 8A. However, except for caudate (*p* = 0.012), the difference in contrast-to-noise ratio between the proposed method and OSEM-PSF was not significant (*p* > 0.05) for cerebellum, frontal gray matter, lentiform nucleus, parietal and temporal lobe.

**Table 3:** *P*-values showing statistical significance using paired *t*-test (*p* < 0.05 than significant) for proposed method by comparing contrast-to-noise ratio of the reconstructed images with FBP, OSEM and OSEM-PSF.

Comparison between	Caudate	Cerebellum	Front GM	Lentiform nucleus	Parietal lobe	Temporal lobe
FBP vs. proposed method	0.0008	0.0013	0.00297	0.0024	0.0023	0.0016
OSEM vs. proposed method	0.0004	0.0033	0.0192	0.0097	0.0077	0.0085
OSEM-PSF vs. proposed method	0.0116	0.0606	0.3762	0.1505	0.4551	0.2653

The degree of contrast-to-noise ratio improvement was both participant and region dependent. For example, in the caudate for P1, the proposed method provided nearly 17% improvement in contrast-to-noise ratio. In contrast, in temporal region for P2, the difference between OSEM-PSF and the proposed method was negligible. The contrast-to-noise ratio obtained by the proposed method in all participants was better than for FBP and OSEM, irrespective of the brain region considered. Consequently, we limit our subsequent comparisons to OSEM-PSF and the proposed method.

#### 3.4.2 Validation against noise, motion and attenuation

Figure 2 shows the results of testing against noise, motion and attenuation artefacts. When low count input data were used, the proposed method outperformed OSEM-PSF in image contrast and signal-to-noise ratio. OSEM-PSF resulted in higher levels of noise compared to the high-count reference image but a good level of structural detail was retained. Noise in images produced using the proposed method did not increase when low count sinograms were used as inputs.

When low count sinograms were combined with simulated motion, (see Figure 2B) OSEM-PSF images were further degraded. While the images generated by the proposed method were also degraded, they were substantially superior to the OSEM-PSF images. The proposed method was able to generate high quality images in the presence of motion provided high count data were used (compare the third column of Figure 2B to the first column of Figure 2A).

When attenuation correction was not incorporated into the sinogram, both OSEM-PSF and the proposed method created similar images which differed from the reference image but preserved brain anatomy (compare Figure 2C to reference images in Figure 2A). Differences included increased signal variation across anatomically similar brain regions, widening of the extracerebral space and image blurring, all of which are known artefacts of non-attenuation corrected PET images. Taking these findings together, Modular GAN appears to provide a valid, high-quality projection from a sinogram to the corresponding PET image.

## 4 Discussion

Analytic and iterative PET image reconstruction methods such as FBP and OSEM are routinely used. Machine learning methods provide new opportunities in medical image reconstruction. We proposed Modular GAN as a deep learning method of mapping a brain sinogram to a PET image. The key benefits of using this method over FBP and OSEM include training with synthetic data, robustness to low count data and participant motion, and production of high quality images when benchmarked against FBP and OSEM. We quantitatively confirmed that structural similarity, PSNR, contrast recovery and contrast-to-noise ratio all improved with the use of Modular GAN. The results suggests that the generated image quality is superior to the current state-of-the-art method, indicating that the proposed method can be beneficial clinically.

The Modular GAN is trained using non-clinical and atlas-based images. Interest in using deep learning to directly reconstruct high PET images from the sinogram is growing (12). Once trained, these methods generate images more rapidly than conventional methods, creating the potential for real time application. Training datasets for direct methods can be created from existing clinical images, or by performing phantom based studies. However, the paired clinical images (consisting of high dimensional features necessary for adequate training) are often unavailable or difficult to attain and phantom studies may lack the feature space of real images. This gap in the literature motivated us to develop a data-driven method that does not require clinical training images.

### 4.1 Comparison between data-driven methods

A key challenge in machine learning is the collection of sufficient training and testing data. In our work, we trained and tested using non-application specific images, and provided verification of the modular GAN for PET image reconstruction based on scanner collected data. Our results suggest that this type of framework where the modular GAN is trained using non-application specific images can be used to perform PET image reconstruction. Previous work has not evaluated DeepPET and cGAN using non-application specific training and testing images.

As we show in Supplementary Figure 5s, the previous methods are unable to reconstruct a high quality image using non-application specific images for training and testing. Whilst we could have provided DeepPET and GAN results in Figures 2, 7, 8, they would not be a fair comparison since they would not perform as well as the modular GAN based on the non-application specific images employed in our work (refer to Supplementary Figure 5s). However, we can make a few comments. We tested cGAN and DeepPET using 13,000 application specific synthetic images and found Module 1 to produce qualitatively superior images. For DeepPET, the images appeared more blurred, while cGAN had better structural information it was unable to reproduce the hotspot (refer to Supplementary Figure 6s). We recommend future studies to evaluate how other machine learning PET image reconstruction methods should be trained and tested using non-application specific images.

We, and others, have SSIM to measure structural similarity between images but this measure has distinct shortcomings when applied to comparisons across studies (40). The SSIM value generated depends on the intensity range in the image, what area or how much of the image is used to compute the value, and whether negative values are used in the SSIM calculation (40). For these reasons, comparisons based on rRMSE provide a better benchmark across studies. Hence while we evaluate SSIM improvements within studies, the metric is not used for comparisons across studies.

AUTOMAP was the first direct reconstruction method proposing automated image transformation using non-clinical images (13). PET images reconstructed using AUTOMAP were inferior in quality to OSEM images. The likely cause of this was reported to be due to conversion to 2D sinograms using the method of single slice rebinning and to mismatched training and test data (41). DeepPET was the next direct PET image reconstruction method to be developed. It used convolutional layers without a fully connected layer within the encoder-decoder framework (15), thereby allowing training using higher resolution phantom data. DPIR-Net added the use of discriminator and perceptual losses to the DeepPET model and required real human PET imaging data for training (42).

The published results of quantitative (using phantom images) and qualitative (using human images) assessment of DeepPET indicate that for phantom images it resulted in 11% smaller rRMSE, 1% larger SSIM, and 1.1 dB higher PSNR than OSEM. For clinical images, qualitatively Deep PET resulted in the loss of structural information compared to OSEM. However, signal-to-noise ratio was larger, possibly because DeepPET produced smoother images. Arguably the smoothing also results in loss of small hotspots in PET images, not apparent in the OSEM image reconstruction. The excessive smoothness and loss of detail was a primary reason for creating DPIR-Net, with the addition of perceptual and Wasserstein distance losses to the framework.

Quantitative assessments using simulated or phantom images have not been published for DPIR-Net. Scanner generated PET images were Radon transformed to a sinogram which was used as the input into the network. DPIR-Net achieved a high SSIM (0.917–0.980,) when compared to the scanner image indicating that DPIR-Net potentially outperforms DeepPET. However, the SSIM improvement over DeepPET was marginal (at best 2%, but mostly less than 1% and no rRMSE calculations were provided). DeepPET produced images which were comparable to OSEM in SNR and SSIM. In contrast, as shown in Figure 4, Modular GAN outperformed OSEM on average by 15% (refer to Table 1).

Recently, an encoder-decoder network was used to demonstrate the feasibility of deep learning to rapidly reconstruct images acquired using a long axial field of view PET (12). Corresponding sinograms and PET images from 80 participants were used for training and the algorithm was compared against the ground truth of scanner generated images (using OSEM with time-of-flight correction) and images reconstructed using DeepPET. The phantom results were not as good as human results, which can be explained by the network having been trained using only human data, with potentially insufficient variability. The SSIM with ground truth high count human test images was 0.90–0.975 compared to 0.87–0.95 for DeepPET. rRMSE was slightly smaller than for DeepPET (<10%), and PSNR was slightly larger (<2%). For low count data, the metrics were worse than for the DeepPET image generated using high count data. Our benchmark NEMA phantom results, refer to Figure 6 and Table 2, showed that Modular GAN outperforms OSEM on every metric provided (10% larger contrast recovery, 21% larger contrast-to-noise ratio). These findings, in addition to qualitative human brain images in Figure 7 and quantitative results in Figure 8, demonstrate the ability of Modular GAN to produce high quality, low noise images, with image contrast better than in OSEM images.

### 4.2 Modular GAN features

GANs are trained via adversarial learning of generator-discriminator networks. Conditional GANs are the state-of-the-art for image-to-image synthesis, such as sinogram to image mapping in this study. We added receptive field constructs to the discriminator to define a relationship for an output activation with an area in the input image. We found this to be critical for the training of Modular GAN. Our loss-convergence plots for receptive fields of 70 × 70, 16 × 16 and 10 × 10 concluded that training convergence was best when the smallest receptive field was used (see Supplementary Figure 4s). Improved training performance was also reported in work conducted by Jaipuria et al. (43), which considered GANs for multi-modal image synthesis.

The use of adversarial loss tends to produce smooth, low contrast images, while non-adversarial and perceptual losses lead to better network performance after training (44). Our improved contrast recovery and contrast-to-noise ratio for the NEMA phantom study (see Figure 6 and Table 2) are likely due to the incorporation of these losses into Modular GAN (see Figure 1). Our finding that contrast-to-noise ratio in human images improved by as much as 20% for Modular GAN compared to OSEM is consistent with the NEMA phantom results, where 14%–30% improvement was achieved for different regions (compare Figure 8 with Table 2). The human contrast-to-noise ratio results are lower than expected from the NEMA phantom findings, possibly because the selected regions of interest contain mixtures of cerebrospinal fluid and white and gray matter.

The design of conditional GANs capable of producing highly stochastic outputs that capture the full entropy of the conditional distributions they aim to model is an open question identified by Isola et al. (18). Studies in multi-modal image synthesis have been conducted, attempting to answer this question (45). Normally, the latent code is provided to the generator through an input layer, i.e., the first layer of a network. However, style GAN departs from this design by omitting the input layer and by starting with a learned constant instead (20). Here, a non-linear mapping network first produces an intermediate latent space, which then controls the generator through adaptive instance normalisation (AdaIN) at each convolution layer. By doing so, spatially invariant style features can be computed from a vector, instead using an image prior.

Noise or motion reduction techniques in PET studies have often relied on information from MRI to compensate for signal loss due to acquisition limitations. However, our proposed method takes a different approach. Inspired by the idea from style GAN, we mixed style-regulating parameters using a non-linear mapping network after the bottleneck layer of the residual network-based encoder using adaptive instance normalisation (see AdaIN in Module 2 of Figure 1). It is worth noting that these style-regulating parameters are not generated using patient images but are instead determined by the additional mapping network, which depends on the data distribution used for training Module 2. This can be evident from the ablation study performed with and without style embedding in Module 2 (refer to Figure 5). The enhancements in PSNR (on average by 4.5%) and SSIM (on average by 4.2%) are particularly noteworthy when style embedding is used compared to the performance of Module 2 without style embedding. Nonetheless, the observed improvement was non-systematic and statistically insignificant, likely attributable to the network architecture of Module 2. Here, latent noise of dimension 1,024 was passed through the FC-Block. How the fully connected layers in the FC-Block influence noise intensities warrants further examination.

The network learns spherical cavities as controlled stochastic variations, but retains global features for the wedge sections (Figure 4D). As such, the network does not make spatially inconsistent mappings. Based on our experiments, this helps with mapping low count data to a high-quality image and motion. However, the network sometimes produces random non-linear artifacts (Figure 2B). Signal attenuation is also not completely recovered for cortical regions in the final reconstructed images (Figure 2C). Nevertheless, results for attenuation were promising when compared to non-attenuated corrected OSEM, especially close to the skull. Because of the “black box” nature of deep learning, the process underlying learning of the attenuation transformation is unclear and requires additional, dedicated experiments; our purpose was to validate the effectiveness of non-clinical training images using GANs.

We note that the number of angular bins for the acquired sinogram is scanner specific. If the model is trained with inputs of the same size as scanner sinogram data (140 × 360), the restricted information within the input domain imposes limitations on the model's ability to learn the underlying sinogram to image mapping. To overcome this limitation, the scanner-based sinograms were reorganised. We observed that while the acquired scanner data sampled sinograms from 0° to 180°, we achieved more favorable outcomes for training Module 1 when sinograms were sampled from 0° to 360°. This choice is guided by the fact that sampling over 360° provided an effective information set in the input domain compared to the 180-degree sampling. This observation can be validated by comparing Supplementary Figures 7sA, sD, where the increased information in the sinogram is evident.

## 5 Conclusion

We propose a two-module GAN-based direct reconstruction of a PET image from a sinogram. Using this framework, we produced PET images of higher quality than those obtained using OSEM and FBP. We showed the network to be robust to noise and motion by reconstructing low dose images close in quality to their high dose counterparts, while the reconstruction of motion-corrupted sinograms also improved with the use of modular GAN. The training of the network using synthetic data and the high quality of image reconstruction that was achieved highlights the future potential for machine learning in medical image reconstruction and synthesis, when domain specific data is limited.

## Data Availability

The raw data supporting the conclusions of this article will be made available by the authors, without undue reservation.
