# Supplementary material for: Modular GAN: positron emission tomography image reconstruction using two generative adversarial networks
Source: Front Radiol. 2024 Aug 29;4:1466498. doi: 10.3389/fradi.2024.1466498 (PMC11425657; doi:10.3389/fradi.2024.1466498)
Supplement: Supplementary file 1 [file Datasheet1.docx]

**Supplementary material 1:**

Training image Source

To mitigate potential bias in the dataset and ensure generalisability during training, the performance of the GAN was evaluated across ten distinct subsets of the dataset. The training dataset consisted of non-clinical images sourced from diverse online repositories. Specifically, it incorporated the Sculptures 6k dataset, comprising a collection of 6340 images. Additionally, the Oxford buildings dataset, encompassing 5062 images procured from Flickr (1, 2), was included. Another dataset utilised was the describable texture dataset, which encompasses a rich assortment of 5640 images showcasing various textures (3). Lastly, the Caltech 256 dataset, featuring a comprehensive assemblage of object images classified into 101 categories, was integrated into the training process (4).

In the preprocessing stage, all images were converted to grayscale. The dimensions of the images were standardized to a height and width of 256 pixels each. Additionally, a padding of 20 pixels was applied along the boundaries of the images. This padding served to maintain the structural integrity and avoid potential information at the edges. These images were forward project using Radon transform to generate the sinograms. As a result of these preprocessing steps, two distinct pairs i.e. image and sinogram were obtained for training the conditional GAN. The selection of images for training was guided by specific parameters that aimed to capture both complexity and variability within the dataset. These parameters were chosen based on the intricate nature of human perception (5). By incorporating complexity and variability, the GAN network was exposed to a diverse range of structures, features and patterns, enabling it to learn the sinogram to image mapping and generalise effectively.

The training images can be downloaded from https://drive.google.com/file/d/1Xjl4Br-SZXPDtq-hc8GGZuaMvsRebAaZ/view

Image complexity parameters

Complexity is a multifaceted concept with varying definitions across different disciplines. In the context of digital images, complexity can be understood as the amount of information required to specify the structure and state of the object being studied. When considering complexity at the pixel level, it relates to factors such as heterogeneity, redundancy, contrast, content and artistic impressions(6). Psychological research has attempted to quantify visual complexity by examining how individuals perceive one image as more complex than another (5). Furthermore, when applying machine learning techniques to analyze images, it has been found that image complexity is influenced by image features described in previous studies (7, 8). This suggests that the perception of visual complexity emerges from the interplay of several dimensions.

In our study, we adopted a comprehensive approach to define image complexity and variability by analysing different parameters. In this lieu, we define average mean symmetrical error (AMSE), contrast per pixel (CPP), entropy, fractal resolution (FD) and number of principal components required to define 95% variability in images (PCA_C). Alongside, ratio of CPP by entropy (C/E) and ratio of average mean symmetrical error by FD (A/F) were also considered. AMSE is the average of the mean squared error between the pixels of an image along its horizontal and vertical axis. CPP is an estimation of the average intensity difference between a pixel and its adjacent pixel. Entropy is a statistical measure of randomness that can be used to characterize the texture of the input image and fractal resolution can be seen as a measure of roughness of the image.

Analysis of complexity in Dataset

Number of images used in different subsets are shown in figure 1s-A. We varied the parameters selectively in different sets with different number of images ranging from 7611 to 28000. AMSE is the crucial parameters but within tradeoff with other factors. Datasets 9 and 8 within a range of AMSE 1086-11450 & 1592-12337 trains the network effectively in comparison with datasets 1 & 2 with large range of 0-15229, 0-9485. Also, dataset 7, 5 and 10 with ranges between 3209-10308, 2997-10757 and 3953-11404 respectively are less effective. Although, dataset 4 has AMSE range close to 9 and 8, (of 1960-13970) yet it is unable to train the network due to limited range of contrast per pixel lying between 8.47 and 20 as shown in figure 1s-F.


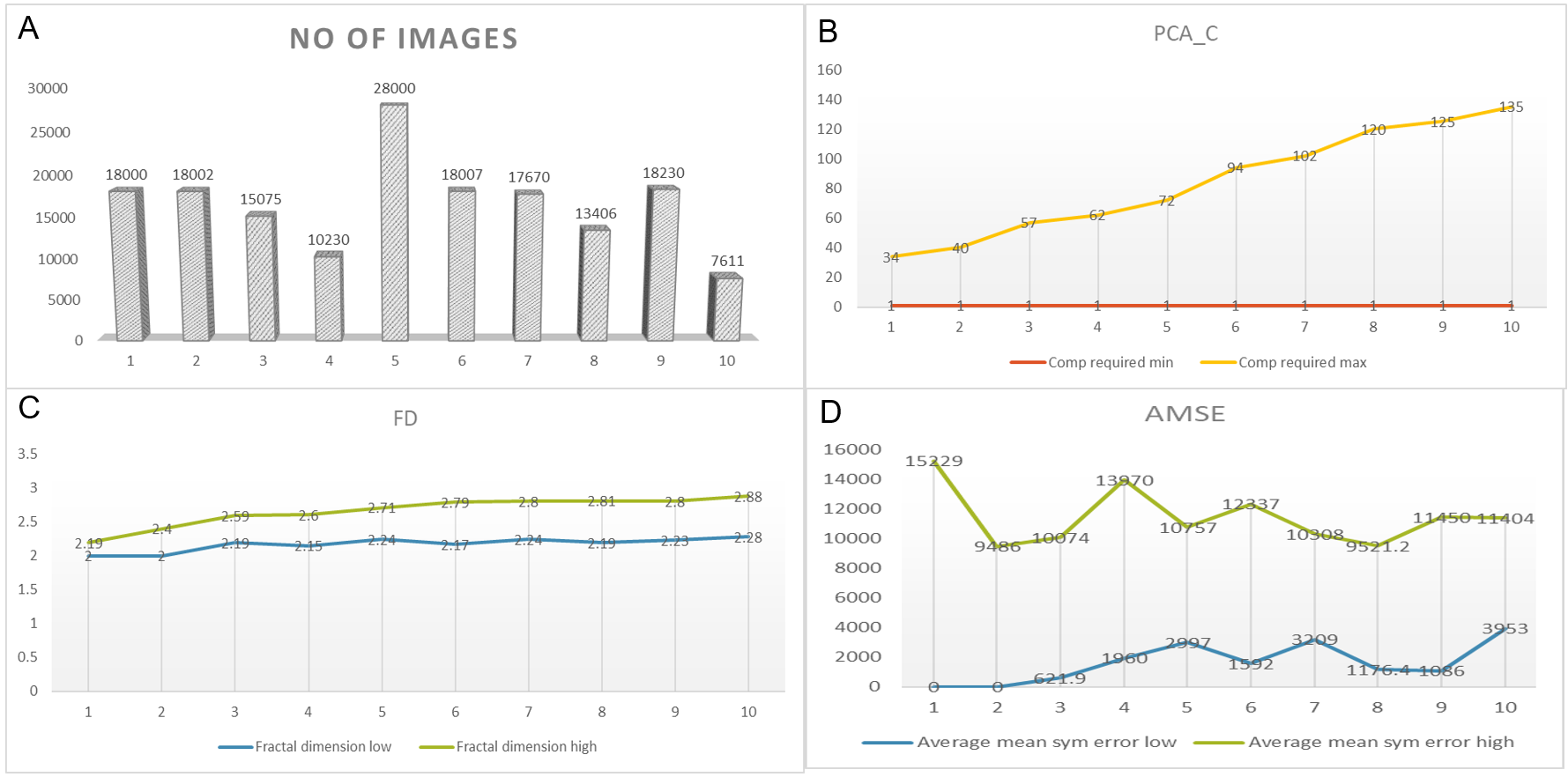


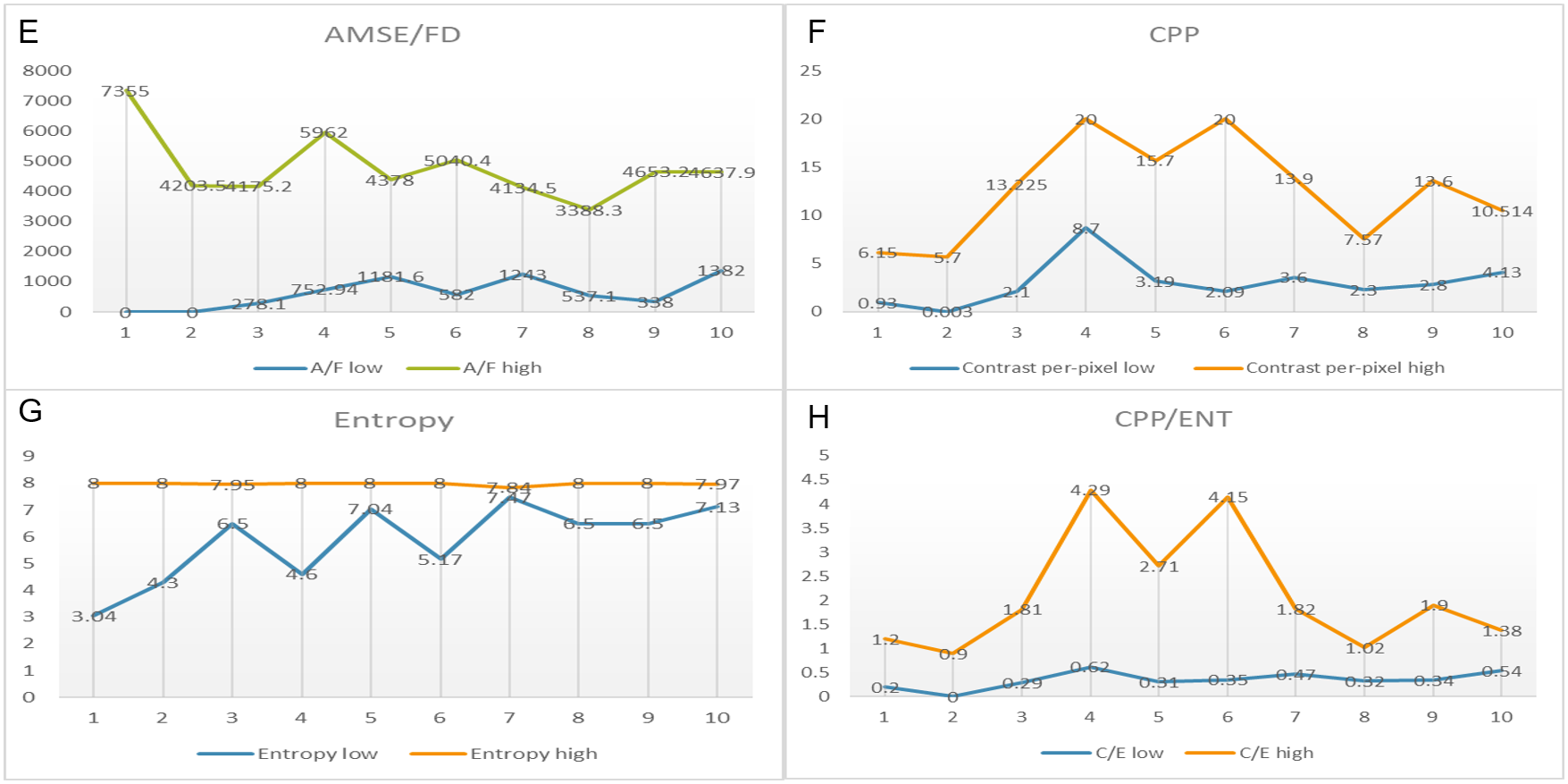


Figure 1s: A-H) Representation of different subsets (on x-axis) with parameters range (y-axis) defining complexity and variability.

Likewise, dataset 9 and dataset 6 has the contrast range of 2.8-13.6 and 2.09- 20.0 respectively with CPP/ENT ratio lying between 0.34 to 1.9 and 0.35 to 4.15 as shown in figure 1s-H. Consequently, this range of CPP/ENT ratio for dataset 9 and 6 proves to be better than other when compared for similarity index of the test images as shown in figure 2s-B. While for the dataset 2, 1 and 10 with range of 0-0.9, 0.2-1.2, 0.54-1.38 lacks it. On contrary dataset7 and 4 has CPP/ENT range of 0.47- 1.82 and 0.62-4.29 that is close to dataset 9 and 8 also deficits similarity. It is due to the fact that in dataset 7 the range of entropy is very close i.e. 7.47-7.84 and for dataset 4 the CPP range is between 8.7 and 20. It signifies that CPP/ENT range of 0.35 to 4.15 is crucial such that CPP and entropy should range from 2.09-20 and 5.17-8 respectively.

Nevertheless, this range of these two parameters decreases the PSNR and increases the MSE. Therefore, to make a trade off, a subset range of these two parameters such that CPP in the range of 2.8-13.6 and entropy in the range of 6.5-8 with CPP/ENT of 0.34-1.9 holds effective significance. Moreover, the datasets 1 and 2 with lower FD range are not suitable though they have large AMSE. Consequently, the range of FD between 2.23 to 2.8 with AMSE/FD ratio range of 338-4653 holds effectivity.

Qualitatively, the comparison with the images generated by training the architecture using different dataset during test phase has been shown in figure 2s-A. Whereas, the five test images are the slices of the different anatomy of brain.


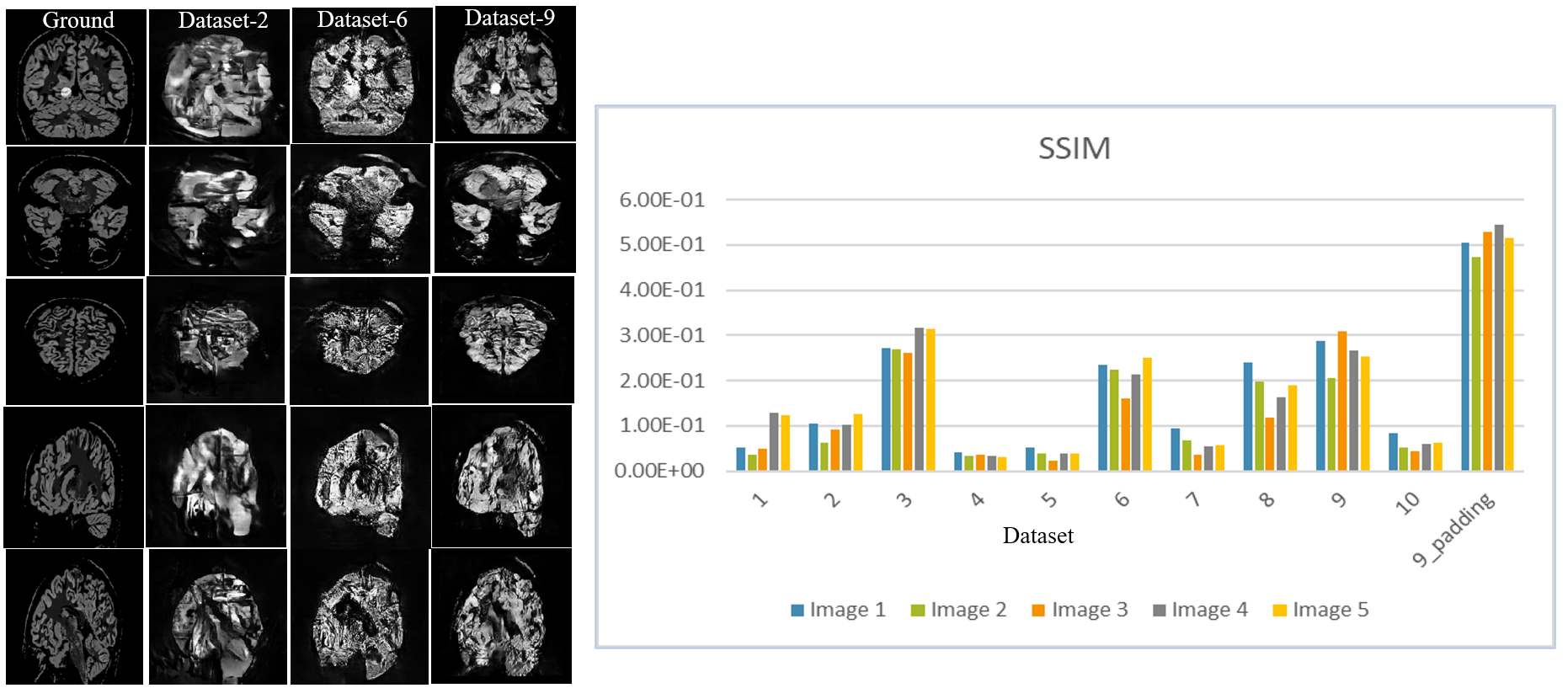


Figure 2s: A) Qualitative analysis of test brain images (1-5) when GAN was trained on different image subsets. Ground truth images are at left. B) Structural similarity observed on the different subsets of dataset for five brain test images is shown on y-axis.

Among the variability parameters AMSE is the essential component in learning the shape/boundaries of the anatomies; however, it is not the only essential component that helps in better training. Textures in the images, which is measured in terms of FD along with AMSE plays a crucial role in learning the shape and textures. We establish this notion by comparing different datasets. For this we compare the SSIM between the generated images and the ground truth. The one with only higher AMSE although tends give the desired shape but it lacks the texture. On contrary, the dataset with higher FD lacks the spatial boundaries.

Moreover, dataset with less images of lower fractal resolution prevents the learning of weights in generator architecture. In this scenario after few iterations the generator loss stops converging. It can be reasoned as generator images tries to fool the discriminator very easily. As such we also noticed that if we select the images with higher AMSE error, than FD of such images becomes low. Therefore, to overcome this limitation we take the composite of two images. i.e. one with higher error and one with higher FD. Consequently, the tradeoff between the FD and the AMSE is essential for desired shape and texture in the generated images. This can be seen from the ratio (A/F). However, this is regardless with the FD or AMSE of the test images. As FD of test brain images is between 2.25 to 2.30. Besides when FD of the training images are placed below 2.3 the results are not as desired.

Likewise, contrast per pixel and entropy are the other two important parameters in the images that controls the training. While the images with only low high entropy are significantly heterogeneous in terms of pixel intensities. Still, the variability in its range between 3 and 8 generates images with effective similarity. Similarly, image datasets with higher CPP images tends to produce images with high mean squared error and low PSNR between the ground the generated image. However, image dataset with a range of low CPP did not prove as good selection too. Consequently, a subset range lying between 4 and14.5 holds significant result. Furthermore, to quest the adequate number of images with in these two range the ratio between CPP and entropy of images is considered. Experiment results reveal that the images between the ratio of 0.3 to 1.9 better trains the network and the generator produce more characteristic images on the test data. Quantitative assessment of these parameters shows that the AMSE, FD CPP entropy and the ratio of A/F and C/E helps is selecting a better dataset for training. Thus, interdependency between these parameters at pixel level is crucial.

**Supplementary material 2:**

Simulated brain images for testing Motion and Noise


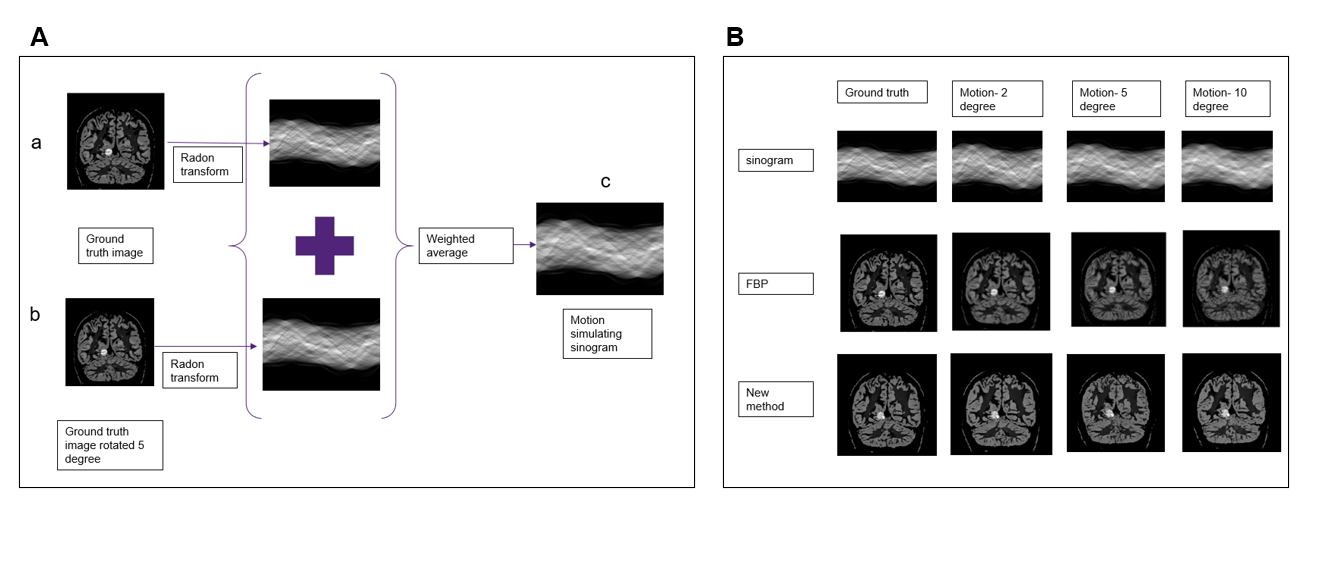


Figure 3s: A) Motion simulating pipeline for PET brain image. B) Top row shows the radon transform of original image in the column one. Column 2-4 shows the weightily added sinograms of original image and rotated image with 2, 5 and 10 degree of rotation. In second and third row the images reconstructed using FBP and developed proposed method are represented for the row one sinograms.


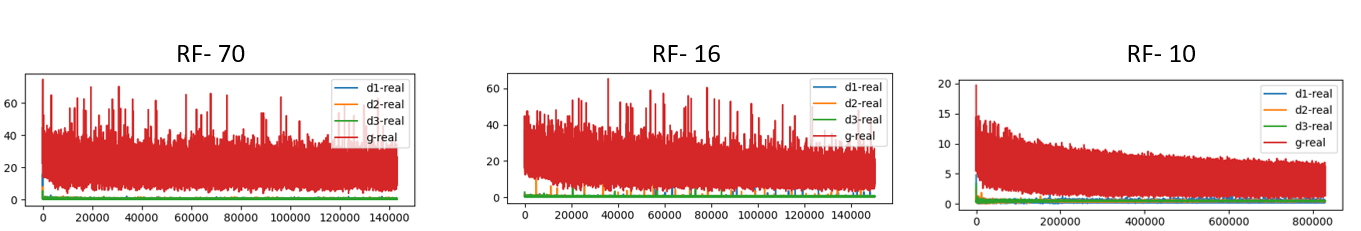


Figure 4s: Loss-convergence plots for receptive fields of $70\times70$, $16\times16$ and $10\times10$


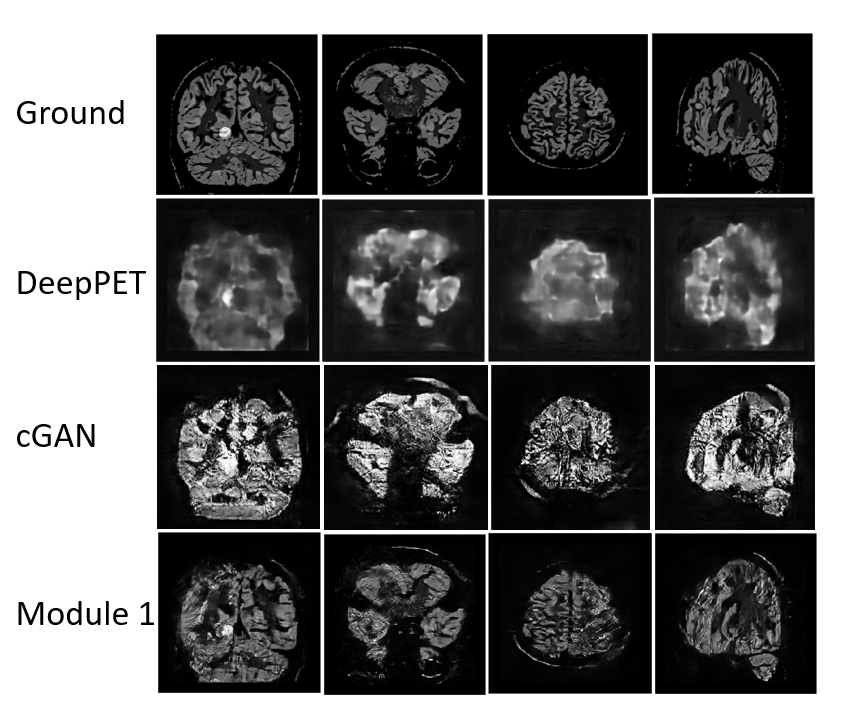


Figure 5s: Qualitative comparison between the generated image quality between DeepPET, image conditional Gan and Module 1 of the proposed method when trained using the non-application specific images.


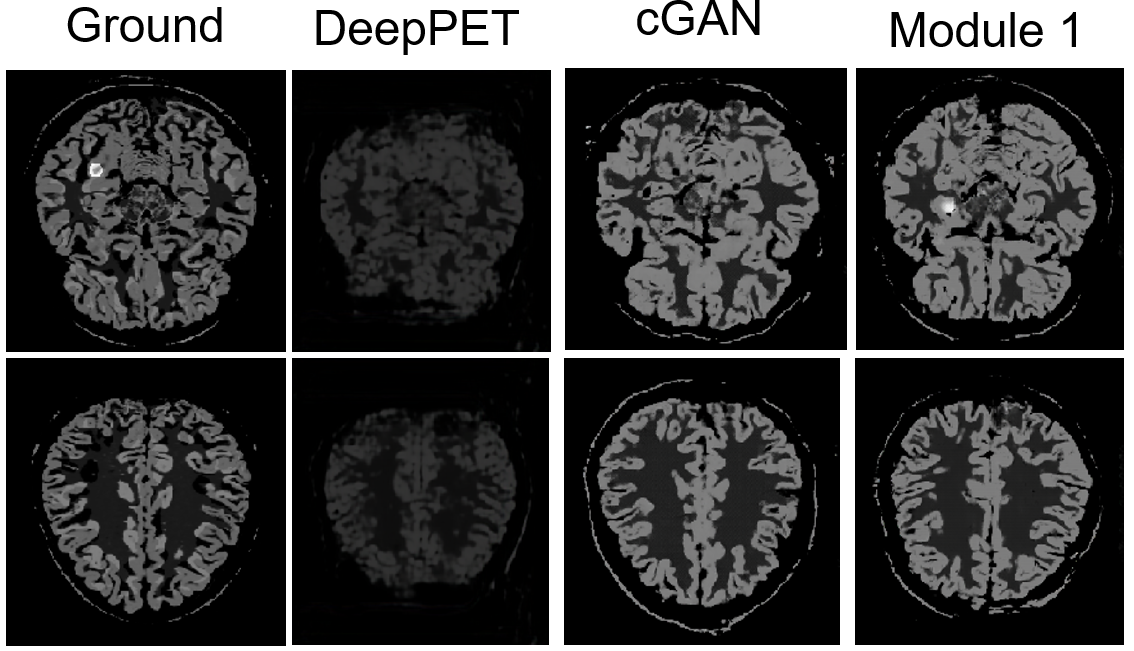


Figure 6s: Qualitative comparison between the generated image quality between DeepPET, image conditional Gan and Module 1 of the proposed method when trained using the application specific images.


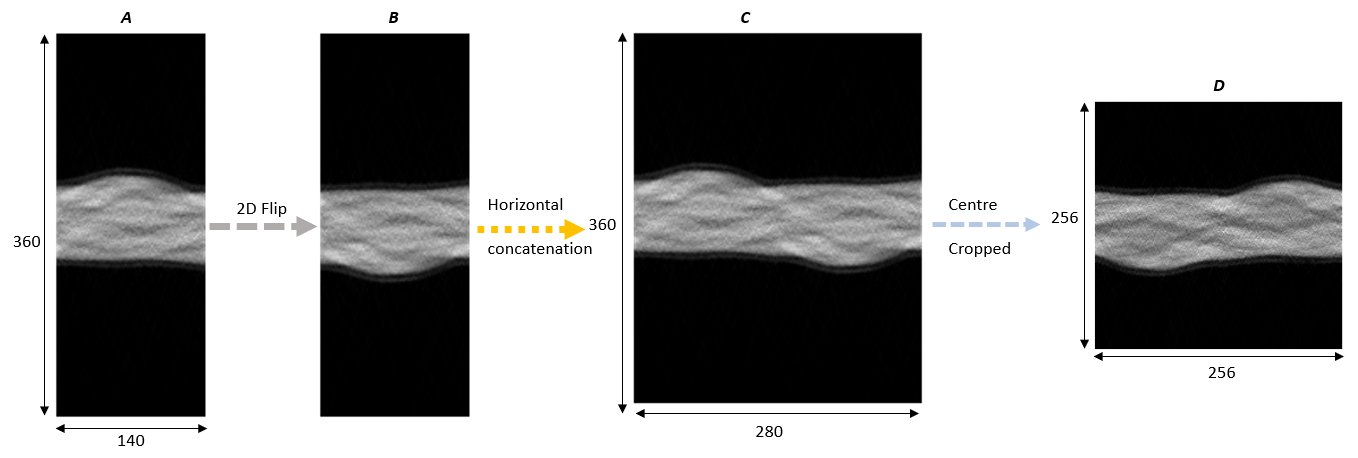


Figure 7s: Two-dimensional time-of-flight Fourier rebinned sinograms from the scanner (A). To increase the sampling of projection angles from 180 to 360 degrees, the sinogram was flipped vertically (B) and then horizontally concatenated with the original sinogram (C). The resultant sinogram was centrally cropped (D) to eliminate void spaces.

**References**

1. Arandjelović R, Zisserman A, editors. Smooth object retrieval using a bag of boundaries. 2011 International Conference on Computer Vision; 2011: IEEE.

2. Philbin J, Chum O, Isard M, Sivic J, Zisserman A, editors. Object retrieval with large vocabularies and fast spatial matching. 2007 IEEE conference on computer vision and pattern recognition; 2007: IEEE.

3. Cimpoi M, Maji S, Kokkinos I, Mohamed S, Vedaldi A, editors. Describing textures in the wild. Proceedings of the IEEE conference on computer vision and pattern recognition; 2014.

4. Griffin G, Holub A, Perona P. Caltech-256 object category dataset. 2007.

5. Hübner R, Fillinger MG. Comparison of objective measures for predicting perceptual balance and visual aesthetic preference. Frontiers in psychology. 2016;7:335.

6. Gartus A, Leder H. Predicting perceived visual complexity of abstract patterns using computational measures: The influence of mirror symmetry on complexity perception. PloS one. 2017;12(11):e0185276.

7. Machado P, Romero J, Nadal M, Santos A, Correia J, Carballal A. Computerized measures of visual complexity. Acta psychologica. 2015;160:43-57.

8. Zanette DH. Quantifying the complexity of black-and-white images. PloS one. 2018;13(11):e0207879.
